# EANM guidelines on the use of [^18^F]FDG PET/CT in diagnosis, staging, prognostication, therapy assessment, and restaging of plasma cell disorders

**DOI:** 10.1007/s00259-024-06858-9

**Published:** 2024-08-29

**Authors:** Cristina Nanni, Christophe M. Deroose, Sona Balogova, Constantin Lapa, Nadia Withofs, Manil Subesinghe, Bastien Jamet, Elena Zamagni, Davide Ippolito, Michel Delforge, Francoise Kraeber-Bodéré

**Affiliations:** 1 Nuclear Medicine, IRCCS Azienda Ospedaliero-Universitaria di Bologna, Bologna, Italy; 2 Nuclear Medicine, University Hospitals (UZ) Leuven, 3000 Leuven, Belgium; 3 Nuclear Medicine and Molecular Imaging, Department of Imaging and Pathology, KU Leuven, Leuven, Belgium; 4 Nuclear Medicine, Comenius University, Bratislava, Slovakia; 5 Médecine Nucléaire, Hôpital Tenon, GH AP.SU, Paris, France; 6 Nuclear Medicine, Faculty of Medicine, University of Augsburg, Augsburg, Germany; 7 Division of Nuclear Medicine and Oncological Imaging, Department of Medical Physics, CHU of Liege, Liege, Belgium; 8 GIGA-CRC in Vivo Imaging, University of Liege, Liege, Belgium; 9 Department of Cancer Imaging, School of Biomedical Engineering and Imaging Sciences, King’s College London, London, UK; 10 Médecine Nucléaire, CHU Nantes, F-44000 Nantes, France; 11 IRCCS Azienda Ospedaliero-Universitaria di Bologna, Istituto di Ematologia “Seràgnoli”, Bologna, Italy; 12 Dipartimento di Scienze Mediche e Chirurgiche, Università di Bologna, Bologna, Italy; 13 Department of Diagnostic Radiology, Fondazione IRCCS San Gerardo dei Tintori, Via Pergolesi 33, 20900 Monza, Italy; 14 University of Milano-Bicocca, School of Medicine, Via Cadore 33, 20090 Monza, Italy; 15 University Hospitals (UZ) Leuven, 3000 Leuven, Belgium; 16 Nantes Université, Univ Angers, INSERM, CNRS, CRCI2NA, F-44000 Nantes, France

**Keywords:** Plasma cell disorders, Multiple myeloma, EANM guidelines, [18F]FDG PET/CT

## Abstract

We provide updated guidance and standards for the indication, acquisition, and interpretation of [^18^F]FDG PET/CT for plasma cell disorders. Procedures and characteristics are reported and different scenarios for the clinical use of [^18^F]FDG PET/CT are discussed. This document provides clinicians and technicians with the best available evidence to support the implementation of [^18^F]FDG PET/CT imaging in routine practice and future research.

## Preamble

The European Association of Nuclear Medicine (EANM) is a professional, non-profit, medical association that facilitates communication worldwide amongst individuals pursuing clinical and research excellence in nuclear medicine. The EANM was founded in 1985. These guidelines are intended to assist practitioners in providing appropriate nuclear medicine care for patients. They are not inflexible rules or requirements of practice and are not intended, nor should they be used, to establish a legal standard of care. The ultimate judgment regarding the propriety of any specific procedure or course of action must be made by medical professionals taking into account the unique circumstances of each case. Thus, there is no implication that an approach differing from the guidelines, standing alone, is below the standard of care. To the contrary, a conscientious practitioner may responsibly adopt a course of action different from that set out in the guidelines when, in the reasonable judgment of the practitioner, such course of action is indicated by the condition of the patient, limitations of available resources or advances in knowledge or technology subsequent to publication of the guidelines. The practice of medicine involves not only the science but also the art of dealing with the prevention, diagnosis, alleviation and treatment of disease. The variety and complexity of human conditions make it impossible to always reach the most appropriate diagnosis or to predict with certainty a particular response to treatment. Therefore, it should be recognized that adherence to these guidelines will not ensure an accurate diagnosis or a successful outcome. All that should be expected is that the practitioner will follow a reasonable course of action based on current knowledge, available resources and the needs of the patient to deliver effective and safe medical care. The sole purpose of these guidelines is to assist practitioners in achieving this objective.

## Definitions

[^18^F]FDG: 2-[^18^F]fluoro-2-deoxy-D-glucose is a radioactive glucose analogue that is injected intravenously and accumulates in areas of high glucose metabolism. These includes normal structures such as the brain and heart, as well as a wide range of tumors, infection and inflammation.

Computed tomography (CT): An ionizing cross-sectional imaging modality that uses x-rays to create three-dimensional images of the body, reflecting variations in tissue density. This allows for adjustment of the PET data for attenuation, facilitating high-resolution visualization of tumors. A PET/CT scan may encompass various types of CT scan, differing based on CT acquisition parameters and the option of using oral or intravenous contrast.

Diagnostic CT scan: A CT scan that typically involves higher X-ray doses than a low-dose CT scan with the option of using oral or intravenous contrast. It is recommended that diagnostic CT scans be conducted in alignment with local consensus protocols or established national guidelines.

Low-dose CT scan: A CT scan to correct for attenuation (CT-AC) and pinpoint the anatomical positions of PET observations (utilizing lowered settings for the X-ray tube’s voltage and/or current). The low-dose CT is not designed for in-depth radiological analysis.

Magnetic Resonance Imaging (MRI): A non-ionizing cross-sectional imaging technique that uses a strong magnetic field and radio waves to excite hydrogen atoms. Upon relaxation, these atoms release energy, which is detected and measured by the scanner and used to generate three-dimensional images of the body. MRI images have excellent soft tissue contrast, which can be tailored to highlight specific tissue characteristics by adjusting various acquisition parameters.

Positron emission tomography/computed tomography (PET/CT): A physical combination of PET and CT which allows sequential acquisition of CT and PET components of the study, whilst the patient remains in the same position.

Whole-body imaging: Imaging field of view from skull vertex to the feet.

Torso imaging: Imaging field of view from skull base to mid-thighs. This scan coverage includes the majority of sites involved in many cancers (standard for both Europe and the USA). If indicated, imaging can be extended to include the brain (skull vertex to mid-thighs).

## Introduction

Multiple myeloma (MM) is a neoplastic disease characterized by the proliferation and accumulation of B-lymphocytes and plasma cells (PCs), which synthesize monoclonal immunoglobulin (M-protein or paraprotein) in the bone marrow (BM) or, more rarely, in extramedullary tissues. Neoplastic B-lymphocytes, originating from the follicular germinal center of the lymph nodes, migrate to the BM where they directly interact with both stromal cells and extracellular matrix [1].

The most crucial cytokine involved in MM growth, both in vivo and in vitro, is interleukin-6. It exerts both a proliferative and anti-apoptotic effect by activating osteoclastogenesis and inhibiting osteoblasts, which leads to bone loss and the development of osteolytic lesions. Neoplastic PCs stimulate BM angiogenesis through production of vascular endothelial growth factor (VEGF) and fibroblast growth factor (FGF). Increased angiogenesis characterizes advanced disease with focal lesions, whilst end-stage disease is characterized by the appearance of a small fraction of highly proliferative plasmablasts, which can cause extramedullary dissemination or plasma cell leukemia [1].

The frequency of MM increases with age, reaching a peak between the sixth to seventh decades with a median age of 65 years; less than 10% of patients are diagnosed between the second to fourth decades. MM is twice as common in males than females, higher in black people than white people and lowest in people from the Asian ethnic group; these differences are likely related to both genetic and environmental factors. The main known risk factors for MM are occupational exposure to pesticides, petroleum and ionizing radiation, and the presence of monoclonal gammopathy of undetermined significance (MGUS) [1].

MM is a much more heterogeneous and complex disease than previously thought, placing it at the boundary between solid and hematological tumors [2]. MM patients can be stratified into two subgroups based upon the prevalent recurring genomic aberrations identified by fluorescence in situ hybridization (FISH):

- Hyper-diploid (H) karyotype: Affects between 40–60% of patients and is characterized by the presence of several trisomies with an overall number of chromosomes ranging from 47 to 75 and either no or rare translocations.
- Non-hyper-diploid karyotype (NH): Characterized by the presence of either less than 46 chromosomes or some translocations of up to 75 translocations of the immunoglobulin heavy chain locus (IgH) on chromosome 14q32 with recurrent partners (particularly on chromosome 4p16.3, 11q13, 16q23 and 6p25) and either deletion or monosomies of chromosome 13. The translocation of IgH commonly causes the over-expression of proto-oncogenes located on partner chromosomes (*FGFR3*, *WHSC1*, *CCND1*, *IRF7/MUM1*, *c-maf*), due to the dislocation of the strong IgH enhancer.

The presence of most chromosomal aberrations in newly diagnosed patients has been proposed as an important prognostic factor with several copy number abnormalities (CNAs), as well as translocations, associated with poorer prognosis, whilst others have no impact [2]. However, it is important to note that the prognostic significance of any evaluated chromosomal aberration should be considered in the therapeutic context, and thus might change over time, since different drugs might affect different genomic backgrounds in different ways. While FISH analysis remains crucial for MM diagnosis due to its accessibility, emerging technologies are mainly utilized in clinical studies. High throughput sequencing identifies a broader array of genomic alterations, such as point mutations, deregulation of gene expression, and copy number alterations (CNAs). However, the prognostic value of these alterations is not yet fully comprehended, though they are expected to be recognized as genomic indicators of more aggressive disease in the future. A notable finding from high throughput sequencing is the detection of genetically diverse subclones within a single patient during any stage of the disease (intra-clonal heterogeneity), which evolve over time and are shaped by treatment. This phenomenon of clonal evolution, observed in various solid cancers and other blood malignancies, could play a significant role in the development of resistance to treatment and the progression of the disease [2].

**Fig. 1:**
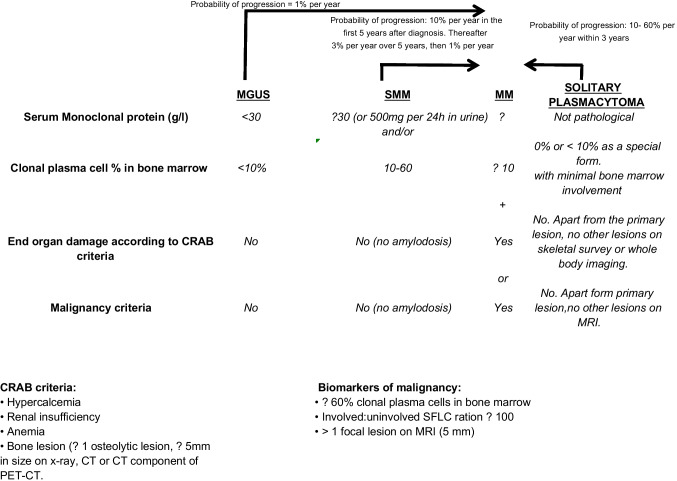
Schematic overview of the diagnostic criteria of IMWG [4]

The common feature of all plasma cell disorders (PCDs) is the production and secretion of an M-protein. The M-protein is mostly an intact immunoglobulin (IgG, IgA or very rarely IgD or IgM) that can be quantified and specified by serum electrophoresis and immunofixation. Serum free light chain (SFLC) production (κ or λ) frequently accompanies the intact immunoglobulin but can be the dominant or exclusively produced paraprotein in 15–20% of patients (light chain MM). FLC are routinely measured in a 24-h urine specimen (Bence Jones protein) but can also be quantified in the serum by immune assays [3]. Although M-proteins can cause organ damage, they primarily serve as markers for disease activity and response monitoring during treatment; in 1–2% of MM patients, no paraprotein is produced, i.e., non-secretory MM. The International Myeloma Working Group (IMWG) produced updated criteria in 2014 for the classification of PCDs [4] (Fig. 1).

BM aspiration and BM biopsy are performed at diagnosis to identify and quantify clonal PC percentage. BM aspiration also allows the identification of specific (high-risk) genetic abnormalities. In combination with levels of serum albumin and β2 microglobulin, the presence or absence of poor-risk cytogenetic features is used to categorize patients into the current prognostic scoring systems, including International Staging System (ISS) and Revised ISS (R-ISS and R2-ISS) [5–7]. Response and disease evolution are scored according to the international uniform response criteria for therapy in MM [8].

The treatment of MM has dramatically changed over the last two decades, changing MM from an acute life-threatening cancer into a chronic, albeit mostly incurable, disease. Although the long-term outcome has significantly improved for most MM patients, this benefit in outcome is associated with long-term treatment and multiple lines of therapy. Historically, the treatment of MM was primarily based on alkylating agents and steroids, but over the last two decades several new drug classes have been introduced. The most important are proteasome inhibitors, immunomodulatory drugs and monoclonal antibodies; the latter compounds are used in various combinations creating several treatment options but with increased complexity in MM management particularly at relapse.

Although international guidelines from the European Hematology Association (EHA) and European Society for Medical Oncology (ESMO) exist, specific factors including age, frailty, comorbidities, previous treatments, drug availability and patient preference have to be taken into account when treating patients with MM [9]; a detailed description of the different treatment combinations is beyond the scope of this paper. During treatment and follow-up in treatment-free intervals, regular MM disease monitoring is primarily performed by sequential measurements of the M-protein, and if indicated, BM aspiration, e.g. measurement of minimal residual disease (MRD) and/or imaging investigations [10].Very recently, a new wave of immunotherapies including chimeric antigen receptor (CAR) T-cells and bispecific antibodies have shown unprecedented therapeutic efficacy in patients with relapsed and refractory MM, which will transform the treatment landscape and further improve the prognosis of MM patients [11, 12]. In addition to treatments that target the underlying clonal PCs, MM patients require additional supportive care to prevent disease and treatment-related complications, which include the management of bone disease, preservation of the kidney function and prevention of infections [11, 12].

### [^18^F]FDG PET/CT

#### Procedure of PET/CT

The board-certified practitioner with overall responsibility for the procedure is governed by national legislation. Further details on qualifications of personnel involved in PET/CT can be found in the EANM procedure guidelines for tumor imaging: version 2.0 [13].

#### Request

The referring physician must provide a request which contains sufficient medical information, such as diagnosis and clinical question(s) to be answered to enable justification of the PET/CT examination.

#### Review of the medical history

Several aspects of the medical history should be reviewed as listed below:

- Myeloma type, known tumor sites, including sites of extramedullary disease.
- Oncological history.
- Current and recent medication, especially antidiabetic medication, corticosteroids, growth factors and sedatives. For therapy evaluation, type and date of last therapeutic intervention is important.
- Current clinical symptoms, e.g., pain, fractures, trauma, night sweat, fatigue, fever.
- Co-morbidities including, chronic kidney disease, allergies, thyroid dysfunction, trauma, fractures, infectious or inflammatory diseases, other tumors.
- Height and body weight; these must be measured accurately to enable standardized uptake value (SUV) measurements.
- Serum glucose.
- Outcomes from additional imaging procedures (notably standard X-rays, CT, MRI, bone scans, and earlier PET/CTs), encompassing acquisition dates, comprehensive reports, and, when feasible, DICOM information from the mentioned analyses for comparative purposes.

- Renal function. Given the high rate of myeloma related kidney disease, any potential use of intravenous (i.v.) contrast requires special attention. In most cases, whole body low-dose CT can be considered sufficient for evaluation of anatomy. If i.v. contrast is to be used, e.g., to enable accurate anatomical delineation of extramedullary disease and relationship to important structures, serum creatinine and estimated glomerular filtration should be measured. Standard assessment of Bence-Jones proteinuria is not mandatory [14]; volume depletion may predispose to acute kidney injury in these patients by enhancing light chain precipitation within the renal tubules. Volume repletion prior to the study is protective if i.v. contrast is required.
- Allergy to i.v. contrast agents. If a PET/CT examination with i.v. contrast is required (see example above), it is important to be aware of any previous contrast reaction(s), which are classified as either idiosyncratic (anaphylactoid) or non-idiosyncratic. Premedication reduces the risk of recurrent anaphylaxis, and if required, the referring physician must indicate the premedication protocol to prepare the patient. For patients with a history of a severe contrast reaction, an unenhanced CT examination is preferred.

#### Patient preparation and precautions

Ideal preparation of patients leads to diminished tracer absorption in regular tissues (such as the kidneys, bladder, skeletal muscle, heart muscle, and brown fat), while enhancing tracer accumulation in the target areas affected by myelomatous deposits and maintaining radiation doses as low as possible (ALARA principle). The appropriate protocols for acquisition are detailed in the EANM procedure instructions for tumor imaging: version 2.0.[13]. A few relevant points are discussed below:

- Patients without diabetes are advised to abstain from eating any food, simple sugars, or beverages, except for plain (unflavored) water, for a minimum of 4 hours prior to the commencement of the [18F]FDG PET/CT scan (relative to the [18F]FDG injection time). Intravenous glucose solutions and parenteral nutrition should be halted at least 4 hours before administering [18F]FDG.
- If using i.v. contrast, adequate prehydration, e.g. consumption of 1 liter of water 2 hours prior to [^18^F]FDG injection, is important to ensure a sufficiently low concentration of [^18^F]FDG in the urine (fewer artefacts) and to minimize the risk of kidney injury.
- Coffee or caffeinated beverages are not recommended because even if “sugarless” they may contain traces of simple carbohydrates and have the potential to induce excitant effects; this may also be the case for “sugar-free” beverages.
- Following the administration of [18F]FDG and during the absorption phase, patients are advised to stay seated or lying down and quiet to limit muscle uptake of [18F]FDG. To reduce brown fat activation, patients should be kept warm, beginning from 30-60 minutes prior to [18F]FDG injection and continuing during the uptake period and image acquisition. Various methods and substances have been tested to decrease brown fat uptake, such as patient warming, administering 5mg of diazepam intravenously 10 minutes before [18F]FDG injection [15], or taking 80mg of propranolol orally 2 hours prior to [18F]FDG injection [16], although outcomes have been inconsistent [17].Patients should void immediately prior to the PET/CT examination to reduce bladder activity.
- Patients need to remain motionless in the PET/CT scanner throughout the exam. Inquiring about claustrophobia when arranging the study can reduce non-diagnostic exams and cancellations and facilitate the planning for premedication. Regular use of sedatives, such as short-acting benzodiazepines, in adult patients is not recommended. Given the high prevalence of bone disease, optimal patient positioning is mandatory. In addition, analgesics can be used to relieve patient discomfort.
- The patient should put their arms alongside their body to enable a complete whole-body examination; support devices, e.g., foam pallets for the arms should be employed whenever feasible to reduce artefacts generated by beam hardening on the spine. Alternatively, patients can be asked to position their hands on their abdomen.

#### Serum glucose levels before [^18^F]FDG administration

The main objectives of patient preparation with at least 4 h of fasting are to ensure low blood glucose level and low insulinemia, as insulin is directly responsible for glucose uptake by non-tumor cells [18]. All further relevant details are outlined in the EANM procedure guidelines for tumor imaging: version 2.0 [13]. Intravenous administration of insulin before [^18^F]FDG injection has been trialed but is yet to have be adopted [19].

#### PET/CT acquisition

Patients should be imaged in the supine position with arms alongside the body. However, special caution must be taken to avoid CT truncation artefacts. If available, an extended field of view (FOV) for both the CT and PET components of the study is recommended; the PET coverage should not deviate from the FOV used for the CT component. The CT and PET acquisitions should be conducted from the skull vertex to the toes (or at least to the knees). Acquisition of the PET component should start from toes and proceed cranially to ensure that the bladder, which fills during imaging acquisition, is as empty as possible after pre-scan voiding; this reduces the risk of misalignment with the CT component due to an enlarged bladder and minimizes scatter and halo artifacts. For a standard step and shoot non-continuous bed motion PET/CT, the time of PET acquisition is approximately 2–3 min per bed position and can be adapted accordingly in continuous motion or large axial FOV PET systems.

For CT, protocols should follow national guidelines and be chosen regarding the objective of the examination according to the supervising board certified practitioner’s suggestion. Whereas whole-body low-dose CT (WB-LDCT) is considered the standard in MM, diagnostic contrast-enhanced scans can be performed in patients with normal kidney function and adequate hydration if applicable [14].

#### Image reconstruction

PET reconstruction (ordered subset expectation maximization, OSEM) should include protocols with and without AC to detect artefacts [13]. When available, time-of-flight information should be used during reconstruction. PET AC, or PET “non-attenuation correction” (NAC), PET, CT, and PET/CT should be displayed on a hospital-wide picture archiving and communication system (PACS), ideally. WB-LDCT can be acquired according to parameters outlined by the IMWG Bone Working Group [20], whilst for diagnostic CT scans, acquisition parameters should be determined according to locally agreed protocols or national guidelines. It is essential to evaluate the images prior to the patient’s departure from the department to confirm the technical adequacy of the PET/CT scan and to determine if further imaging is required or if there is an immediate need to communicate with the referring doctor, for instance, in cases of fractures or spinal cord compression.

## Radiological tests

The main purpose of imaging in MM is to identify sites of bone disease that necessitate starting treatment. Myeloma-related bone disease is defined as the presence of one or more osteolytic bone lesions (≥ 5 mm) attributable to an underlying clonal PCD [4]. The mainstay of radiological imaging in MM for many years has been the skeletal survey, comprising radiographs of the axial and appendicular skeleton (chest, spine, humeri, femora, skull, and pelvis) including symptomatic regions; up to two thirds of newly diagnosed patients with myeloma (NDMM) have osteolytic bone disease visible on plain radiographs [21]. However, despite its low cost and widespread availability, the advent of whole-body imaging, i.e., WB-LDCT, whole-body MRI (WB-MRI) and [^18^F]FDG PET/CT, has exposed the low sensitivity of radiographs for the detection of osteolytic bone disease [22] and highlighted its inability to assess BM based disease. Accordingly, international guidelines [3, 4, 23–25] now recommend whole-body imaging for the assessment of myeloma-related bone disease, although the choice of first line imaging modality varies across guidelines and clinical indications.

The IMWG [23] recommends WB-LDCT as first line for suspected MGUS and suspected smoldering myeloma (SMM) and either WB-LDCT or [^18^F]FDG PET/CT for suspected MM; WB-MRI is recommended if there are inconclusive findings on prior whole-body imaging. WB-MRI is recommended as first line for suspected solitary plasmacytoma (SP) of the bone, whilst [^18^F]FDG PET/CT is first line for suspected extramedullary SP. The ESMO guidelines [24] confirm that WB-LDCT is the gold-standard for diagnosis of osteolytic disease, whilst WB-MRI or [^18^F]FDG PET/CT are appropriate alternatives. The European Myeloma Network (EMN) [3] recommend WB-LDCT as first line for suspected SP or MM and pelvic-spinal MRI (PSMRI)/WB-MRI if there is no osteolytic disease evident on WB-LDCT or in cases of suspected spinal cord compression. [^18^F]FDG PET/CT is considered a suitable alternative to WB-LDCT if the CT component of the study permits accurate assessment of the skeleton from vertex to knees and includes the arms. The National Institute for Health and Care Excellence guidelines [25] recommend WB-MRI as first line for suspected MM and either WB-LDCT or [^18^F]FDG PET/CT as alternatives for patients with NDMM. WB-MRI and [^18^F]FDG PET/CT are recommended for patients with non-secretory MM or extramedullary SP. Ultimately, the choice of whole-body imaging modality is based upon availability of scanners and scanner capacity as well as resources, which includes specialist experience of reporting these complex imaging studies.

### Whole body-low dose-CT (WB-LDCT)

CT provides superior sensitivity over plain radiographs for the detection of osteolytic lesions within the skeleton. The generation of three-dimensional high-resolution images overcome the superimposition of anatomical structures inherent in plain radiographs and enables earlier detection of bone loss, particularly in the spine and pelvis as well as other areas poorly assessed by radiographs [26]. Pathological fractures, sites ‘at-risk’ of fracture, and both paramedullary disease (PMD) and extramedullary disease (EMD) can also be assessed with CT. CT is relatively cheap compared to other whole body imaging modalities, is widely available, quick to perform (< 1 min), and does not require any change in patient position during image acquisition. However, the high radiation dose associated with conventional CT, relative to the skeletal survey, has led to the development of WB-LDCT protocols extending from skull vertex to knees (without intravenous iodinated contrast), which are associated with much lower radiation doses (3.2–4.8 mSv) without compromising diagnostic accuracy [4]. WB-LDCT guidelines regarding image acquisition, image reconstruction and image interpretation have been developed [20] and are applicable to the low-dose CT component of [^18^F]FDG PET/CT.

### Whole body MRI (WB-MRI)

WB-MRI has superior soft tissue contrast to WB-LDCT and can demonstrate BM involvement, either focal or diffuse, prior to cortical bone destruction [27]. As a result, the IMWG [4] state that > 1 (i.e., 2 or more) focal lesion measuring ≥ 5 mm in size on MRI is a myeloma defining event (MDE). Guidelines for image acquisition, interpretation and reporting of WB-MRI in myeloma have been published [28] and recommend diffusion-weighted imaging (DWI), a functional imaging sequence, as part of a core clinical protocol. DWI reflects the free random motion of water, which becomes increasingly restricted with higher percentages of malignant PC infiltration, and manifests as high signal intensity on DWI sequences; this enables accurate differentiation from normal fatty BM and underpins the higher sensitivity of DWI for both focal and diffuse BM infiltration, compared to conventional MRI sequences [29]. Generic limitations of MRI include claustrophobia, which may be exacerbated by using large body coils for WB-MRI, non-MRI conditional implants and devices, and renal failure, if using gadolinium-based contrast agents. Limitations specific to WB-MRI, include greater cost and lower availability compared to WB-LDCT; PSMRI can be used in cases where WB-MRI is unavailable albeit with the potential to miss disease (10%) lying outside of this FOV [30]. WB-MRI is also time consuming, taking between 30–50 min to complete, and can be especially problematic for patients suffering from myeloma-related bone pain.

## Indications

### [^18^F]FDG PET/CT for initial diagnosis and staging

Diagnosis of MM according to the IMWG criteria [4] highlights the importance of whole-body imaging owing to its superiority over the skeletal survey in detecting myeloma-related bone disease [31–33]. [^18^F]FDG PET/CT enables the detection of individual or multiple osteolytic bone lesions > 5 mm, a CRAB criterion. In addition, it allows tumor cells and the tumor microenvironment to be identified based on their propensity to consume higher levels of glucose compared to normal bone cells/tissues [33].

### Disease detection in symptomatic multiple myeloma

[^18^F]FDG PET/CT has a sensitivity of 90% and a specificity between 70 to 100% for detecting various MM lesions [34–36]:

1. Focal lesions (FLs)—foci of [^18^F]FDG uptake greater than background (acquired on 2 successive images), either with or without bone osteolysis,
2. Paramedullary disease (PMD) and diffuse BM involvement—soft-tissue lesions contiguous with bone involvement and with variable glucose metabolism and maximum standardized uptake values (SUV_max_) [34–39], and
3. Extramedullary disease (EMD) [40, 41]- abnormal soft tissue without contiguous bone involvement,
4. Diffuse BM involvement—diffuse glucose uptake in the axial skeleton (greater than liver uptake and either heterogeneous or homogenous) and the disease may extend to the periphery.

The IMPeTUs interpretation criteria was proposed by the Bologna group to standardize the interpretation of PET in MM (Table 1). They showed that using the Deauville criteria (a 5-point standardized internationally recognized visual interpretation scale) to identify the number of FLs, and characterize the involvement of PMD, EMD, and diffuse BM disease improved the inter-observer interpretation and reproducibility, albeit with persistent variability in the interpretation of skull lesions [42].

**Table 1:** IMPeTUs Criteria for [^18^F]FDG PET/CT reporting in Multiple Myeloma [42]

Lesion type	Site	Number	Grading
Diffuse	Bone marrow “A” if hypermetabolism in limbs and ribs		5-PS
F (Focal)	S (skull) SP (spine) Ex-Sp (extra-spine)	X_1_ (None) X_2_ (*N* = 1 to 3) X_3_ (*N* = 4 to 10) X_4_ (*N* > 10)	5-PS
L (Lytic)		X_1_ (None) X_2_ (*N* = 1 to 3) X_3_ (*N* = 4 to 10) X_4_ (*N* > 10)	
Fr (Fracture)	At least one		
PM (Para-medullary)	At least one		5-PS
EM (Extra-medullary)	At least one	N/EN (Nodal/Extranodal)*	

*For nodal (N) disease: *C* Cervical; *SC* Supraclavicular; *M* Mediastinal; *Ax* Axillary; *Rp* Retroperitoneal; *Mes* Mesentery; *In* Inguinal; For extranodal spread (ENS): *Li* Liver; *Mus* Muscle; *Spl* Spleen; *Sk* Skin; *Oth* Other)

*5-PS* Deauville 5-point scale

NDMM patients have intra- and inter-patient tumoral heterogeneity reflected by variable [^18^F]FDG PET/CT patterns [43–46]. In the French prospective multicentric IMAJEM study performed on 134 NDMM patients, [^18^F]FDG PET/CT was normal at diagnosis for 9% of patients, 33% showed FLs with a median number of three FLs (0 to > 10) and a median FL SUV_max_ of 4.1 (range 1.5–28.4), 9% had diffuse BM involvement, 49% had combined diffuse infiltration and FLs, and 10% had EMD, [41]. In the more recent international prospective multicentric CassioPET study performed on 268 NDMM patients, [^18^F]FDG PET/CT was normal at diagnosis for 20% of patients, 67% had FLs with a median SUV_max_ of 6.1 (range 1.9- 48.5), 48% had diffuse BM infiltration and 8% had EMD. Moreover, PMD was described for the first time in the CassioPET population and was present in 18% [39]. Approximately 10–20% of NDMM patients are [^18^F]FDG PET/CT ‘false negative’ at baseline, a phenomenon known to be due to low hexokinase-2 expression [40, 41, 43, 47]. These patients cannot be monitored after therapy by [^18^F]FDG PET/CT imaging.

The sensitivity of [^18^F]FDG PET/CT for symptomatic MM is comparable/less than pelvic-spinal MRI (PSMRI) [41, 48, 49]. The first direct comparison of these two imaging modalities, albeit in a small series, revealed that [^18^F]FDG PET/CT sensitivity was lower than PSMRI for diffuse BM involvement but it did allow additional FLs to be detected, in particular, those outside of the the MRI FOV [48]. Results from the prospective IMAJEM study published in 2017 compared conventional PSMRI and [^18^F]FDG PET/CT at initial diagnosis and after therapy; 94.7% of PSMRI-scanned patients and 91% of [^18^F]FDG PET/CT patients were positive, demonstrating equivalent sensitivity [41]. More recently, a retrospective comparative study of 46 NDMM patients reported that WB-MRI detected bone disease with a higher per patient sensitivity compared to [^18^F]FDG PET/CT (91.3% vs. 69.6%). Interestingly, the clinical treatment decisions for these patients were very similar and either modality was appropriate for initial staging.[50]. Finally, we and others [51, 52] recently showed that WB-MRI with DWI improves the sensitivity for FL detection, which is higher than [^18^F]FDG PET/CT, especially in SMM patients at initial diagnosis.

### Disease detection in solitary plasmacytoma

[^18^F]FDG PET/CT has a demonstrable benefit for SP patients as it allows additional lesions to be detected with greater sensitivity and specificity compared to PSMRI [53]. A comparison of [^18^F]FDG PET/CT to PSMRI on a single center cohort of 24 SP patients reported sensitivity, specificity, and positive and negative predictive values of 98% and 93%, 99% and 94%, 93% and 84% and 99% and 98%, respectively. In addition, [^18^F]FDG PET/CT identified SP lesions outside the PSMRI FOV, especially extramedullary SPs. In a second example [54], a retrospective study of 43 patients diagnosed with SP showed that [^18^F]FDG PET/CT was diagnostically superior to PSMRI: initial [^18^F]FDG PET/CT identified at least 2 hot lesions in 23% of patients, whilst PSMRI only identified 16% of patients.

### Disease detection in SMM and MGUS

[^18^F]FDG PET/CT imaging has proven utility in SMM [4]. An initial study of 188 SMM patients assessed with [^18^F]FDG PET/CT [55], where a positive [^18^F]FDG PET/CT was defined an abnormal increased uptake (> 1 FL and/or diffuse BM uptake) and/or evidence of osteolytic bone destruction on CT, reported that [^18^F]FDG PET/CT was positive in 74 patients (39%), and negative in 114 patients (61%). In a prospective study of 120 SMM patients, [^18^F]FDG PET/CT was positive in 16% of patients, with eight patients having one FL, three patients with two FLs, six patients with > 3 FLs and two with diffuse BM involvement [56].

According to updated data of the Southeastern Minnesota cohort (with a long-term follow-up) adverse risk factors for progression of MGUS to active MM include an M-protein ≥ 15 g/L and an abnormal SFLC ratio in patients with non-IgM MGUS. Patients with two risk factors showed a significantly higher progression rate to MM (30% over 20 years) compared to patients with no (7%) or one (20%) risk factor [57]. Therefore, there is likely requirement to perform imaging in patients with high risk MGUS, but to date, prospective data regarding the diagnostic performance of whole-body imaging in this setting are lacking. A recent observational retrospective study assessing [^18^F]FDG PET/CT in 338 patients with MGUS [58] reported that 30 patients had MM bone lesions (15 in the initial study and 15 in the follow-up). The monoclonal component rate emerged as the main predictor of a positive [^18^F]FDG PET/CT in adjusted multivariate regression analysis.

### Prognostic value of pre-treatment [^18^F]FDG PET/CT

#### Symptomatic MM

Recent advances have been made in risk stratification, especially thanks to gene expression profiling and cytogenetic data. However, the sensitivity of these tests is somewhat limited due to the single-site nature of BM biopsy sampling and disease spatial heterogeneity. This last point was eloquently shown by Rache et al. [44]: a high-risk genomic alteration identified in a single FL may be unaltered (i.e., germline wild-type) at other locations, emphasizing that single site BM biopsies do not necessarily represent the entire BM milieu [44]. Several [^18^F]FDG PET/CT characteristics have been identified as possible high-risk biomarkers and could be used to define high-risk NDMM patients.

Initial large prospective studies in NDMM patients demonstrated that baseline derived PET parameters of > 3 FLs, an FL SUV_max_ > 4.2 and EMD were associated with poorer progression free survival (PFS) and overall survival (OS) [40, 41, 59]. More recently, we published the combined analysis of 227 NDMM patients from two separate French and Italian phase III trials [60]. Using a multivariate analysis including treatment arm, R-ISS score, and the presence of EMD and bone SUV_max_, we identified that only bone SUV_max_ (*p* = 0.016) was an independent prognostic factor, and the OS threshold was 7.1. The large prospective CASSIOPET study of NDMM patients demonstrated for the first time the negative prognostic value of PMD and reaffirmed the positive effect on PFS of a normal [^18^F]FDG PET/CT at baseline [39, 61]. This is consistent with data published by Rache et al. in 2018 showing that large FLs are a strong independent factor for poor prognosis in NDMM [44, 45]. A study combining transcriptomic analyses using RNA sequencing with [^18^F]FDG PET/CT of a sub-group of 139 NDMM patients from the CASSIOPET study showed that [^18^F]FDG PET/CT-negative patients also had significantly reduced hexokinase-2 (HK2) expression, but also showed enriched HK2 expression in a subgroup of patients with a low level of bone disease [47]. Moreover, positive [^18^F]FDG PET/CT profiles displayed two distinct signatures: elevated expression of proliferation genes or high expression of the glucose transporter GLUT5 and lymphocyte antigens. Lower PFS was independently associated with PMD and IFM15, a well-characterised high-risk MM gene signature, and patients with both biomarkers were at very high risk of disease progression.

Recently, Rasche et al. reported a spatial-longitudinal whole-exome sequencing based on 140 samples collected from 24 MM patients during up to 14 years [46]. Applying imaging-guided sampling they observed three evolutionary patterns, including relapse driven by a single-cell expansion, competing/co-existing sub-clones, and unique sub-clones at distinct locations. Whilst they did not find the unique relapse sub-clone in baseline FL(s), a close phylogenetic relationship was found between baseline FLs and relapse disease, highlighting FLs as hotspots of tumor evolution. In patients with ≥ 3 FLs on [^18^F]FDG PET/CT at diagnosis, relapse was driven by multiple distinct sub-clones, whereas in other patients, a single-cell expansion was typically seen (*p* < 0.01).

#### Solitary plasmacytoma (SP)

A retrospective study by Fouquet et al. examined the association of [^18^F]FDG PET/CT and the SLFC ratio transformation risk from of SP to MM [54]. Of 43 SP patients, 48% had an abnormal SFLC value, 64% had an abnormal SFLC ratio at diagnosis, 33% of patients had ≥ 2 FLs on initial [^18^F]FDG PET/CT and 20% had ≥ 2 FLs on initial MRI. At follow-up (median 50 months), 14 patients transformed to MM with a median time-to-multiple myeloma (TTMM) period of 71 months. At diagnosis, the risk factors significantly associated with TTMM included having ≥ 2 FLs on [^18^F]FDG PET/CT, an abnormal SFLC ratio, and involved SFLC value, and to a lesser extent at completion of treatment, a lack of normalization of involved SFLC value. Additionally, incomplete normalization of involved SFLC value, [^18^F]FDG PET/CT or MRI at the conclusion of treatment also contributed, albeit to a lesser extent. Multivariate analysis demonstrated that an abnormal initial involved SFLC value (OR = 10; 95% confidence interval (CI), 1.0–87.0; *P* = 0.008) and [^18^F]FDG PET/CT findings (OR = 5; 95% CI, 0–9; *P* = 0.032) independently correlated with shortened TTMM. In a distinct cohort comprising sixty-two SP patients who underwent [^18^F]FDG PET/CT prior to treatment, Bertagna and colleagues showed that tumor size was notably larger in patients with [^18^F]FDG avid lesions compared to non [^18^F]FDG avid disease on PET [62]. Twenty-nine patients progressed to MM (average period 18.3 months), with a higher likelihood observed among those with avid lesions. In addition, TTMM was notably shorter in patients with bone SPs, SPs demonstration [^18^F]FDG uptake, and when the SUV_max_ was elevated [62].

#### SMM and MGUS

To date, no large study has assessed the prognostic value of [^18^F]FDG PET/CT in MGUS patients. However, in SMM, [^18^F]FDG PET/CT has demonstrated its prognostic usefulness, even though the IMWG diagnostic criteria [4] indicates that osteolysis is deemed mandatory for considering a positive FL on [^18^F]FDG PET/CT as a criterion for starting therapy. A unique cohort of 122 SMM patients evaluated via [^18^F]FDG PET/CT, revealed that the likelihood of progressing to symptomatic MM within two years (without therapy) was 75% for those with positive [^18^F]FDG PET/CT findings (with or without osteolysis) vs. 30% for patients with a negative [^18^F]FDG PET/CT [55].

Another prospective study involving 120 patients showed a similar two-year progression rate from SMM to symptomatic MM, with 58% of patients showing a positive [^18^F]FDG PET/CT (all without evidence of underlying osteolysis) vs. 33% of those with a negative [^18^F]FDG PET/CT scans [56]. It’s worth noting that these studies were published after the most recent IMWG criteria for MM diagnosis [4], and so FLs without osteolysis are not yet considered as MDEs. WB-MRI remains the preferred imaging modality for SMM as recommended by the IMWG.

### Therapy assessment and MRD (MM)

[^18^F]FDG PET/CT is able to distinguish between metabolically active MM lesions and inactive fibrous residual osteolytic lesions, with an earlier and higher rate of scan normalization than MRI after therapy initiation [33, 41, 63, 64]; the IMWG recommend [^18^F]FDG PET/CT as the preferred imaging technique to evaluate and monitor metabolic response to therapy in MM [23, 33]. This statement was confirmed by the consensus panel recommendations of the 2021 EHA-ESMO Clinical Practice Guidelines and the EANM Focus 4 expert consensus recommendations based on the landmark studies carried out by the Nantes and Bologna groups [9, 40, 41, 65–67].

Among patients with conventionally defined complete response after treatment, a persistent positive [^18^F]FDG PET/CT predicts a less favorable outcome (30-month PFS, 78.7% for negative patients vs. 56.8% for positive patients) [33, 41, 66–69]. In addition to the presence of high-risk features at diagnosis included in the ISS, patient prognosis is also defined by the depth of treatment response [7, 70]. In conjunction with serum and urine M-protein measurements and SFLC value, BM PC percentage and imaging (in case of extramedullary SP), the IMWG uniform response criteria for MM defines response categories as follows: minimal response, partial response, very good partial response, complete response and stringent complete response [70].

Recent therapeutic strategies have led to high rates of complete response, defined as negative immunofixation on the serum and urine (absence of a measurable monoclonal protein) and disappearance of any extramedullary plasmacytomas and < 5% PCs in BM aspirates [70]. Consequently, the IMWG consensus refined the response criteria and included the assessment of MRD using next-generation flow cytometry or sequencing, both able to identify MRD with high sensitivity [70]. A meta-analysis confirmed that BM MRD negativity, defined as the absence of clonal PCs by next-generation sequencing (NGS) and/or next-generation flow cytometry, was associated with improved PFS and OS in cohorts of MM patients included in clinical trials, i.e., the Spanish myeloma group PETHEMA/GEM, the French IFM 2009 and CASSIOPEIA trials [71]. Therefore, MRD assessment is now included in all ongoing clinical trials, but it is yet to be performed in routine clinical practice due to the limited availability of the test and the absence of therapeutic implications.

Focal active disease resistant to treatment can be detected outside the site of BM sampling in patients with MRD negativity, in cases of patchy BM involvement and/or EMD [44, 45, 64, 72]. EMD, a strong risk factor for adverse outcome, can be detected in 5–11% of MM patients at diagnosis and in over 20% at relapse during the course of the disease, with increased detection rates observed after the introduction of [^18^F]FDG PET/CT into the imaging workup of MM [7, 40, 41, 64, 66, 67, 69, 70, 73–76]. Therefore, the IMWG and expert consensus recommend performing post-treatment [^18^F]FDG PET/CT as a complementary tool to MRD assessment to assess for active EMD [65, 70, 77]. Although the prevalence of NDMM patients with negative MRD and positive post-treatment [^18^F]FDG PET/CT is low (5–7%), it may be a clinically relevant predictor of a higher risk of early relapse [41, 64, 78]. On the other hand, the combination of negative MRD and negative post-treatment [^18^F]FDG PET/CT scans confirms complete eradication of the tumor both inside and outside the BM, which is associated with longer PFS [64]. Post-treatment [^18^F]FDG PET/CT normalization is an independent factor predictive of better PFS and OS, even if its definition slightly differs between studies [33, 41, 64, 66, 68, 69, 72].

The IMWG consensus released in 2016 outlines a negative post-treatment [18F]FDG PET/CT as the complete disappearance of all previous areas of [18F]FDG uptake identified at baseline or on preceding [18F]FDG PET/CT scans, or a reduction in intensity to below that of the mediastinal blood pool SUV or below that of surrounding normal tissue [70]. Prior to the introduction of the IMPeTUs criteria by Nanni et al. in 2016 [79], various methods for interpreting [18F]FDG PET/CT scans were employed in the literature.The Italian expert panel showed a high interobserver agreement for all the IMPeTUs criteria at all time points (staging, post-induction and end of therapy) [42]. Among the Deauville scores (DS) tested (DS 2–5) for the main parameters (BM, FLs and EMD), DS 4 provided the highest agreement among all the reviewers, especially for BM assessment [42]. Zamagni et al. tested the prognostic significance of the DS to assess response to therapy with [18F]FDG PET/CT at the end of therapy (pre-maintenance) of NDMM patients from two trials (IMAJEM and EMN02/HO95) [67]. They showed that a complete metabolic response, defined as a DS 1–3 in BM and FLs previously involved, including EMD and PMD, was an independent predictor of PFS and OS. We recommend the use of the DC and IMPeTUs classification for therapy response assessment, proposed by Zamagni et al. (Table 2) [67], although further external validation in additional prospective studies is required prior to formal integration into the IMWG response criteria.

**Table 2:** IMPeTUs Classification for therapy response assessment [67]

Complete metabolic response	Uptake ≤ liver uptake (DS 1–3) in the BM, and previously involved FLs, PMD and EMD (if applicable)
Partial metabolic response	Decrease in number and/or activity of BM and previously involved FLs, PMD and EMD (if applicable) but with persistent lesion(s) with uptake > liver activity (DS 4 or 5)
Stable metabolic disease	No significant change in BM uptake, and previously involved FLs, PMD and EMD (if applicable) compared with baseline
Progressive metabolic disease	New FLs, PMD or EMD (if applicable) compared with baseline consistent with MM

Abbreviations: *BM* Bone marrow; *DS* Deauville score; *EMD* Extramedullary disease; *FL* Focal lesion; *PMD* Paramedullary disease; *MM* Multiple myeloma

When [^18^F]FDG PET/CT is performed during or after therapy, the 2023 EANM FOCUS 4 expert consensus group agreed to report additional items detailed in Table 3 [65]. The interpretation of the CT component of the study is an essential part of the PET/CT report. According to the IMWG, progressive disease is defined as the appearance of new osteolytic lesion(s), ≥ 50% increase from nadir in the sum of the products of the maximal perpendicular diameters of > 1 lesion, or ≥ 50% increase in the longest diameter of a previous lesion > 1 cm in short axis [70]. Importantly, the IMWG states that, unless there is disease progression, no change in treatment can be recommended based on post-treatment imaging results only [23] (Table 4).

**Table 3:**
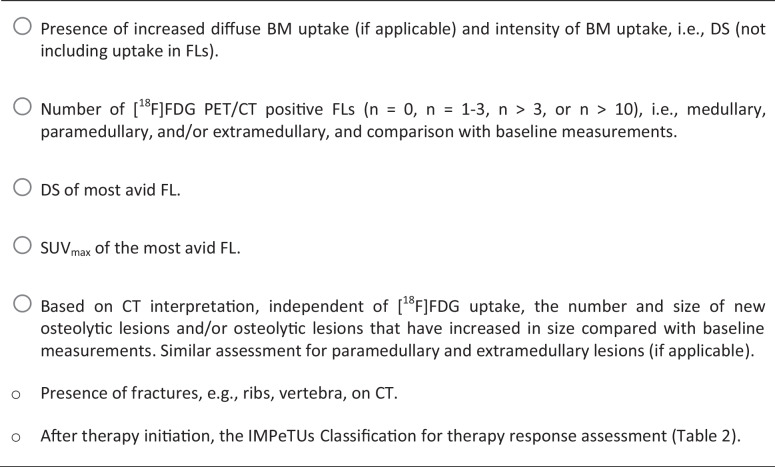
Checklist of items to be reported during or after therapy adapted from Nanni et al. [42, 79]

Abbreviations: *BM* Bone marrow; *DS* Deauville score; *FL* Focal lesion; *SUV*_*max*_ Maximum standardized uptake value

**Table 4:**
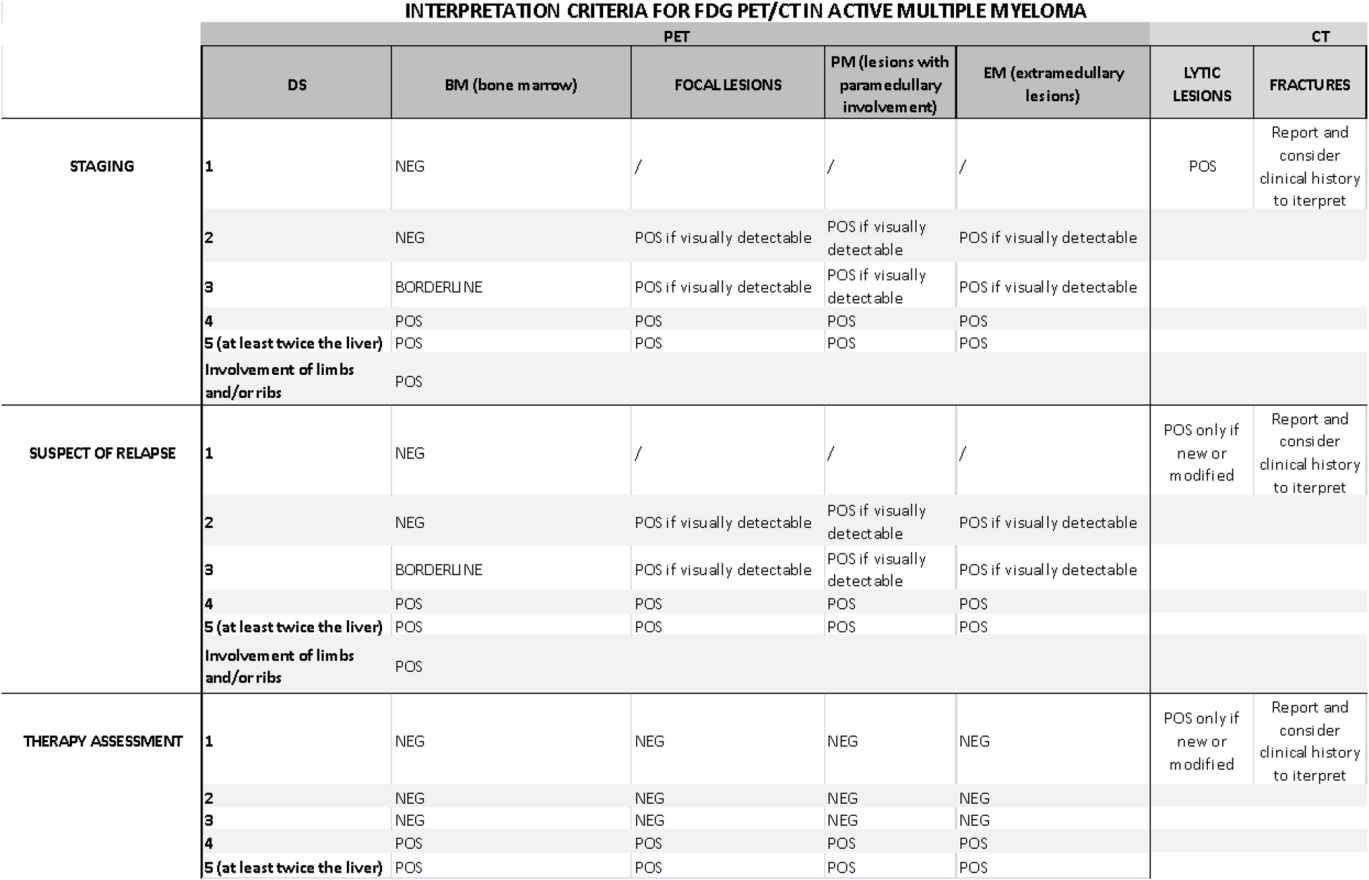
Summary table of the interpretation criteria for [^18^F]FDG PET/CT in multiple myeloma

*DS* Deauville score; *Visually Detectable* A lesion with an uptake higher than local background in 2 or more adjacent slices

There is no expert consensus on the optimal sequence of post-treatment [^18^F]FDG PET/CT scans: interim PET (after induction), end-of-therapy PET (pre-maintenance) and/or during maintenance [65]. [^18^F]FDG PET/CT normalization can be seen as early as at day 7 post-induction and the rate of normalization increases with treatment over time at the end of induction, post transplantation, and maintenance [33, 68]. The DS of sites of disease on [^18^F]FDG PET/CT performed at the end of therapy, before maintenance, shows prognostic significance [67–69]. Zamagni et al. showed that persistence of FLs with SUV_max_ > 4.2 after induction and post-transplant was associated with shorter time to progression [40, 66]. Volumetric measurements using total metabolic tumor volume (TMTV) and total lesion glycolysis (TLG) are potentially informative variables, but standardization of tumor delineation and validation is required before clinical implementation [65].

Although post-treatment [^18^F]FDG PET/CT is a strong prognostic tool, it may not be appropriate for assessment of metabolic response to therapy if the baseline [^18^F]FDG PET/CT is negative, which can occur in 10–20% of patients with NDMM [40, 41, 43, 47, 61, 66–69, 80]. In the Total Therapy population TT4-6, 62% of patients had FLs on baseline [^18^F]FDG PET/CT with a greater percentage in GEP70 high-risk patients [68]. Interestingly, Davies et al. showed that the outcome of patients achieving complete metabolic response was similar to patients without FLs at baseline [68]. The IMWG recommend [^18^F]FDG PET/CT for baseline imaging to enable comparison as part of response assessment [23]. If the baseline [^18^F]FDG PET/CT is negative or if MRI only was performed at baseline, the IMWG recommends performing a WB-LDCT at the end of treatment (before maintenance) which will be used for comparison during follow up [23]. Lastly, it is important to mention that novel immunotherapeutic strategies, e.g., T-cell–redirecting bispecific antibodies, may be responsible for immune-related phenomena and potential pitfalls in [^18^F]FDG PET scan interpretation [81].

### Relapse detection (MM)

The appearance of new plasmacytoma and/or osteolytic bone lesion is one of the criteria defining relapse (or refractory MM) [70] and both the IMWG and EHA-ESMO Clinical Practice Guidelines recommend WB-LDCT (or a localized CT in case of symptoms) when relapse (or progressive disease) is suspected [9, 23]. PSMRI or WB- MRI is recommended in cases of negative or inconclusive WB-LDCT [9, 23]. Imaging using [^18^F]FDG PET/CT is optional; however, it might be of interest for the distinction between active and non-active (non-viable) MM FLs and for the detection of EMD, more prevalent at relapse and adversely affecting both time to progression and OS [9, 33, 82]. In the PETHEMA/GEM2012MENOS65 trial, 14 patients experienced disease progression despite undetectable MRD, six of whom had no detectable M-protein and BM infiltration but had extramedullary plasmacytomas on [^18^F]FDG PET/CT [83]. At relapse, the number of FLs, the presence of EMD and SUV_max_ on [^18^F]FDG PET/CT are associated with PFS but more data are needed to validate their prognostic significance [82, 84–86].

### Follow-up (MM)

Both EHA-ESMO Clinical Practice Guidelines and the IMWG recommend imaging follow-up of MM, including [^18^F]FDG PET/CT if available [9, 23]. The IMWG recommends yearly follow-up using [^18^F]FDG PET/CT in patients with a positive post-treatment [^18^F]FDG PET/CT who are at higher risk of early progression [23, 33, 41, 66–68]. When MRD status is available (currently only in clinical trials), the EHA-ESMO Clinical Practice Guidelines recommend yearly follow-up [^18^F]FDG PET/CT in BM MRD-negative patients to confirm extramedullary MRD negativity; CT or MRI are recommended when symptomatic [9]. The same imaging technique should be used at each stage of follow-up to enable comparison [23].

For SPs, due to a risk of progression to active MM (60% for bone SP and 20% for extramedullary SP within 3 years), the IMWG recommends yearly follow-up for the first 5 years with the same imaging technique used at diagnosis [4, 23]. For SMM, yearly follow-up with PSMRI or WB-MRI is recommended for at least 5 years (depending on risk factors); [^18^F]FDG PET/CT can be used if the MRI is not feasible [23].

## Pet interpretation and reporting system

### [^18^F]FDG PET/CT

MM is a complex disease to interpret on [^18^F]FDG PET/CT imaging. As such, criteria exist to enable standardization of [^18^F]FDG PET/CT reporting both at diagnosis/relapse and for therapy assessment. Furthermore, many PET derived parameters retain a prognostic significance to risk stratification and need to be recognized and reported. It is worth noting that: i) the proposed criteria are entirely visual to minimize the influence of different technologies on scan interpretation; ii) incorporating target-to-background (TBR) ratio measurements to support visual interpretation is deemed acceptable. Semiquantitative indices are determined using a region of interest (ROI) with a radius of ≥ 3 cm placed in the central portion of the liver, away from its edges, and a second ROI completely encompassed within the lumen of the aorta, such as the aortic arch, with precautions taken to avoid vessel wall edges or calcified areas, for the mediastinal blood pool; iii) these criteria have been validated on images reconstructed using OSEM (Ordered Subset Expectation Maximization) algorithms, thus neither time-of-flight nor other algorithms optimizing signal-to-noise ratio should be employed when applying them [42, 79].

The Deauville criteria (5-point scale), the basis for visual interpretation:

- DS 1 = No uptake at all
- DS 2 =  ≤ mediastinal blood pool uptake
- DS 3 =  > mediastinal blood pool uptake, ≤ liver uptake
- DS 4 =  > liver uptake (at least 10% more)
- DS 5 =  >  > liver uptake (at least twice)

At staging, variables to include in the report are:

1. Metabolic state of the BM including the presence of hypermetabolism in ribs and limbs, defined as homogenous or heterogeneous diffuse uptake of the pelvic-spinal-peripheral skeleton > liver uptake.
2. Number and site of FLs on PET, with or without osteolysis on CT. An FL is defined as a visually detectable focal increase in [^18^F]FDG uptake > surrounding BM uptake, located in the skeleton (excluding sites of physiological tracer uptake) on 2 or more adjacent images, with or without osteolysis on CT.
3. Presence of PMD, defined as soft tissue extension contiguous with bone involvement.
4. Presence and site of EMD, defined as abnormal soft tissue without contiguous bone involvement.
5. Presence of fractures on CT.

Variables should be reported according to the IMPeTUs criteria (Table 1).

During or after treatment, therapy assessment is defined as follows [67] (Table 2):

1. Complete metabolic response: Uptake ≤ liver uptake (DS 1–3) in the BM and previously involved FLs, PMD and EMD (if applicable).
2. Partial metabolic response: Decrease in number and/or uptake in BM and previously involved FLs, PMD and EMD (if applicable) but with persistent lesions with uptake > liver uptake (DS 4 or 5).
3. Stable metabolic disease: No significant change in uptake in BM and previously involved FLs, PMD and EMD (if applicable) compared with baseline.
4. Progressive metabolic disease: New FLs, PMD or EMD compared with baseline consistent with MM.

### Interpretation issues / pitfalls

There are several factors to consider when encountering metabolically active bone lesions on [^18^F]FDG PET/CT:

1. A significant proportion of patients with MM present with anemia, which may manifest as an increase in BM uptake, reflecting a compensatory mechanism. This diffuse increase in BM uptake firstly, significantly reduces contrast resolution on PET and the ability to detect co-existent FLs, and secondly, makes it difficult to differentiate from diffuse BM infiltration. A DS ≥ 4 in the BM and the involvement of BM in limbs increase the probability of BM infiltration [42].
2. The metabolic profile of MM is heterogeneous and with variable [^18^F]FDG uptake; 10–20% of patients have absent [^18^F]FDG uptake at staging despite histological confirmation of BM infiltration and/or osteolytic lesions on CT. Despite this, a normal [^18^F]FDG PET/CT is associated with better outcomes than those with a positive [^18^F]FDG PET/CT in line with the well-known phenomenon of increased biological aggressiveness associated with increased glucose metabolism [61]. Consequently, the sensitivity of [^18^F]FDG PET/CT is variable and the interpretation of a single post-therapy [^18^F]FDG PET/CT examination in the absence of baseline [^18^F]FDG PET/CT examination carries the risk of inaccurate assessment response to therapy.
3. Interpretation of early focal BM uptake in the absence of osteolysis on CT is difficult, particularly in those with low glucose metabolism or anemia; it is good practice to consider focal uptake in two or more adjacent images.
4. Recent bone fractures can cause false positive [^18^F]FDG uptake and can take at least one month to normalize, if related to trauma alone.
5. Osteosynthetic material can degrade image quality and result in false positive uptake at the bone-osteosynthetic material interface either related to ongoing bone healing or possibly bone infection.
6. Differentiation of EMD from an unrelated non-malignant pathology, e.g. inflammation, can be difficult and in cases of uncertainty targeted imaging or biopsy should be considered [42].
7. Given that the median age for newly diagnosed multiple myeloma (MM) is approximately 70 years, interpreting findings can be challenging. Degenerative changes in the skeleton may present abnormalities that resemble MM lesions. For instance, recent Schmorl nodules, which are in direct contact with the intersomatic disk and have an osteosclerotic rim, can exhibit uptake on CT images. Additionally, significant focal inflammatory uptake may be observed in tendons, osteophytes, and arthrosic degeneration. In such cases, low-dose computed tomography (LDCT) images can be helpful for accurate assessment.

## PET/CT with tracers other than [^18^F]FDG

[^18^F]FDG is the most studied tracer for imaging PCDs, but false negative observations can occur [43], which has spurred interest in developing other PET tracers. An initial report in 19 patients using the amino acid tracer L-[methyl-^11^C] methionine ([^11^C]MET) showed low uptake in normal BM, high uptake in FLs and additional lesions detected compared to CT in nearly all patients and heterogenous uptake in osteolytic lesions in pretreated patients; in 2 patients, [^35^S]methionine uptake was 5 to 6 times higher in myeloma cells compared to normal BM, demonstrating increased uptake of this amino acid in malignant PCs [87]. In the largest (retrospective) series comparing [^18^F]FDG PET/CT and [^11^C]MET PET/CT, FLs were detected in 47 out of 78 patients (60%) with [^18^F]FDG PET/CT and in 59 patients (76%) by [^11^C]MET PET/CT (*p* < 0.01), resulting in additional disease detection in 12 patients (15%); the inter-reader agreement was also higher for [^11^C]MET PET/CT than for [^18^F]FDG PET/CT [88]. A recent systematic review of head-to-head comparison studies of these 2 tracers reported a difference in patient level sensitivity of > 10% in favor of [^11^C]MET in 5 of the included studies involving 194 patients, with no study showing a benefit for [^18^F]FDG [89]. In a prospective pilot study in patients with treated myeloma negative on [^18^F]FDG PET/CT, [^11^C]MET PET/CT showed focal uptake in osteolytic lesions in 5 out of 7 patients (71%) [90]. These data show that [^11^C]MET PET/CT in selected cases, could be considered as an alternative for patients with negative [^18^F]FDG PET/CT. The main drawbacks of [^11^C]MET are its limited availability (dependent on nearby cyclotron), the limited number of patients scanned per batch and resultant high cost. Alternative amino acid tracers labelled with [^18^F] that could overcome these drawbacks include [^18^F]fluoroethyl-L-tyrosine ([^18^F]FET) [91] and anti-1-amino-3-[^18^F]-fluorocyclobutane-1-carboxylic acid ([^18^F]FACBC) [92].

C-X-C motif chemokine receptor 4 (CXCR4) is an alternative molecular target normally expressed on blood cells and highly overexpressed on myeloma cells [93, 94]. Treatment with anti MM drugs can substantially reduce (bortezomib) or increase (dexamethasone, doxorubicin) expression in MM cell lines or CD138 + patient-derived PCs. The most studied PET tracer targeting CXCR4 is [^68^ Ga]Ga-PentixaFor. Initial studies showed a substantial positivity rate in MM patients (71%) [95]. Comparison with [^18^F]FDG PET/CT has shown mixed results, with an initial small series showing only moderate benefit [96]. A more recent prospective study in NDMM shows significantly higher detection rates with [^68^ Ga]Ga-PentixaFor PET/CT (28/30; 93%) than [^18^F]FDG PET/CT (16/30; 53%) together with a better correlation of quantitative imaging metrics with clinically relevant biomarkers of end organ damage and tumor burden [97]. A similar, albeit retrospective study, in 34 NDMM patients showed higher disease extent in 23 patients (68%) with [^68^ Ga]Ga-PentixaFor PET/CT compared to only two patients (6%) with [^18^F]FDG PET/CT [98]. [^68^ Ga]Ga-PentixaFor PET/CT changed disease stage in 14/34 (41%) patients and were associated with significantly higher median TBRs (5.7 vs. 2.9). In a mixed cohort of 113 MM patients, a positive [^68^ Ga]Ga-PentixaFor PET/CT scan showed high SUV_max_ (~ 19) and TBR (~ 15) Furthermore, [^68^ Ga]Ga-PentixaFor could be used as a theranostic tracer to select patients for CXCR4-targeted radionuclide therapy [95–99]. The first clinical results with lutetium-177 or yttrium-90 based CXCR4 radionuclide therapy in patients with good target expression demonstrated by CXCR4 PET support the further development of this treatment strategy [99].

[^11^C]acetate has been shown to depict the tumoral burden better than [^18^F]FDG in a series of 64 NDMM patients, with detection of BM involvement in 60 patients (94%) by [^11^C]acetate vs. 42 (66%) by [^18^F]FDG [100]. Another study in 35 patients with this tracer showed higher sensitivity to discriminate MM from SMM or MGUS (85% vs. 58%) [101]. Other metabolic tracers including [^11^C]choline or [^18^F]fluoromethylcholine have only been studied in a very limited number of patients.

Molecular imaging of molecular targets targeted by current MM therapeutic regimens could be done with labelled monoclonal antibodies, e.g., targeting CD38 with [^89^Zr]-DFO-DaratumumabProspective, multi-center studies are needed to validate these preliminary data from these novel tracers before their use can be recommended on a routine basis [102].

## Metabolic tumor volumes, radiomics and machine learning

Recent publications have addressed the potential significance of volume-based PET-derived features such as TMTV and whole-body TLG (wbTLG), reflecting total FL tumor burden at diagnosis in MM. The results are conflicting, and the utility of volume-based features is still to be determined. Analysis of two separate prospective European phase-III trials using a Random Survival Forest approach, revealed among all image features and clinical/histopathological parameters collected, that TMTV and wbTLG had less prognostic importance than others, especially BM SUV_max_ or textural features (TF), which is in disagreement with previous published studies [103]. In a large cohort of patients enrolled in Total Therapy 3A, the American Little Rock team showed wbTLG > 620 g and TMTV > 210 cm^3^ at baseline were independent prognostic factors for PFS and OS although the method for segmentation of bone disease requires scrutiny [104]. A retrospective study including 185 patients with NDMM [105] showed that high baseline TMTV (> 56 cm^3^) and wbTLG (> 166 g) values independently predicted both worse PFS and OS but it should be noted that patients’ ages were not homogeneous, so treatments received were likely incomparable [105]. Moreover, the important difference of TMTV prognostic cut-offs values found in these two studies is questionable. High initial TMTV and wbTLG values appear to predict worse PFS and OS in other small retrospective mixed studies but unfortunately with heterogeneous cohorts of patients who received various therapies making it difficult to draw robust conclusions [106, 107]. Statistical analyses used in the aforementioned studies were heterogeneously performed often without external validation. Finally, in a large recent retrospective study of 203 patients with NDMM aiming to investigate the prognostic impact of metabolic heterogeneity (MH), an MH-SUV_max_ lesion (estimated using the area under the curve of the cumulative SUV volume histogram) showed more prognostic relevance than that from a lesion with the largest MTV [108]. The PFS and OS rates were significantly lower in the high-MH-SUV_max_ group than in the low-MH-SUV_max_ group whereas high MH-SUV_max_ retained independent prognostic power on multivariate analysis. Even among patients with high TMTV, those with high MH-SUV_max_ tended to show poorer prognosis than those without. Patients with high MH-SUV_max_ and high-risk cytogenetic abnormalities showed dismal outcomes.

Data published concerning radiomics in MM are scarce up to date. Tumor heterogeneity, as described at the cellular level, could probably be partly captured through medical image analysis, especially using PET-based images. This type of image analysis, often referred to as “radiomics”, has gained significant interest in the past few years with several studies underscoring the potential of TF. The high number of TF extracted from a radiomics approach advocates the use of adapted statistical analysis given the high-dimensional nature of the problem and the associated risk of overfitting with low-complexity models. In this respect, a Random Survival Forest approach outperformed more conventional approaches for prognostic purposes [109].

The potential prognostic value of [^18^F]FDG PET-derived radiomics at baseline in NDMM has been explored for the first time recently in the aforementioned combined analysis of two independent prospective European phase-III trials using a Random Survival Forest approach [103]. Among all image features and clinical/histopathological parameters collected, radiomics were not retained in the final prognosis model based on Random Survival Forest and set by only three features but belonged to the most predictive variables. Further investigations exploring the potential prognostic value of TF in MM using the Random Survival Forest approach are going to be led soon in a larger cohort of patients included in the multicenter international CASSIOPET study [39].

In smaller cohorts, studies have shown the potential of radiomics to enhance radiological assessment of both focal and diffuse patterns of MM on CT [110]. Another study showed the potential of radiomics to detect BM MM infiltration on CT scans of patients with low bone mass [111]. Furthermore, using [^18^F]FDG PET/CT image-based radiomics, Jin et al. demonstrated satisfactory diagnostic performance when classifying MM and bone metastases from various solid tumors [112]. Research has also indicated that a logistic regression-based machine learning method might outperform other methods for determining high-risk cytogenetic status in MM [113]. Finally, in a small cohort of 39 patients, Milara et al. reported on the positive correlations between PET positive cases and TF related to heterogeneity. These included: Entropy, Variance, Short Run Emphasis, High Gray Level Run Emphasis, Short Run High Gray Level Emphasis and Complexity. In contrast, TF related to homogeneity like Energy, Gray Level Non-Uniformity, Low Gray Level Run Emphasis, Long Run Low Gray Level Emphasis and Run Length Variance showed negative correlation with PET positivity [114]. It should be noted that the main problem in using such systems in hematology is data mutability. Further, how inter- and intra-laboratory variability could be addressed and mitigated remain current roadblocks. The application of artificial intelligence in MM is still in a preliminary phase.

## Data Availability

Data sharing: not applicable.

## Declarations

### Liability statement

This guideline summarizes the views of the EANM Oncology and Theranostics Committee. It reflects recommendations for which the EANM cannot be held responsible. The recommendations should be taken into context of good practice of nuclear medicine and do not substitute for national and international legal or regulatory provisions.
